# Preferences for Online and/or Face-to-Face Counseling among University Students in Malaysia

**DOI:** 10.3389/fpsyg.2018.00064

**Published:** 2018-01-31

**Authors:** Kah P. Wong, Gregory Bonn, Cai L. Tam, Chee P. Wong

**Affiliations:** ^1^School of Medicine and Health Sciences, Monash University Malaysia, Subang Jaya, Malaysia; ^2^King Fahd University of Petroleum and Minerals, Dhahran, Saudi Arabia

**Keywords:** online counseling, face-to-face counseling, online therapy, e-therapy, mental health

## Abstract

Increasingly, online counseling is considered to be a cost-effective and highly accessible method of providing basic counseling and mental health services. To examine the potential of online delivery as a way of increasing overall usage of services, this study looked at students’ attitudes toward and likelihood of using both online and/or face-to-face counseling. A survey was conducted with 409 students from six universities in Malaysia participating. Approximately 35% of participants reported that they would be likely to utilize online counseling services but would be unlikely to participate in face-to-face counseling. Based on these results, it is suggested that offering online counseling, in addition to face-to-face services, could be an effective way for many university counseling centers to increase the utilization of their services and thus better serve their communities.

## Introduction

Although traditional face-to-face counseling is the method preferred by most professionals, a large portion of those who could benefit from counseling services do not in fact seek them out. This study looked at preferences among students for face-to-face vs. online counseling and found that a significant proportion of university students in Malaysia would prefer to receive mental health counseling online. Given the overall underutilization of counseling services, it is argued that online delivery of counseling services should be considered as an alternative means of reaching many who remain untreated.

Most people, at some point in their lives, face adjustment difficulties or other mental health challenges. Studies in Malaysia have estimated that between 9.6 and 35% of the population; around 2.8–10 million individuals, could benefit from, but are not receiving, mental health support (Crabtree and Chong, 2000; Chong et al., 2013). Similarly, the World Health Organization estimates that 14% of the global population suffers from some form of mental, neurological, or substance use disorder, and 75% of those in need do not receive any treatment (World Health Organization [WHO], 2016). Despite the availability of on-campus support at most institutions, usage of mental health services among university students appears to be comparably low. Lustig et al. (2006), for example, found that approximately 70% of university students who could benefit from mental health counseling did not utilize available services.

There are many reasons why individuals might hesitate to seek traditional face-to-face counseling (Barak et al., 2008; Gilat et al., 2011). Particularly in Asian societies, stigma associated with mental health problems appears to deter many from seeking professional help (Eisenberg et al., 2007; Al-krenawi et al., 2009; Heflinger and Hinshaw, 2010). Studies have also found that Asians are generally not as comfortable with self-disclosure and are less likely to psychologize their problems compared to those from western societies (Kutcher et al., 2009; Youssef et al., 2014; Haroz et al., 2017). Given the strength of such cultural barriers, qualities inherent in online counseling such as relative anonymity and physical distance could make it an attractive option for many who would otherwise remain untreated (Chester and Glass, 2006; Centore and Milacci, 2008; Rodda and Dan, 2014).

### Online Writing Counseling

A number of researchers have used popular online chat applications such as Whatsapp and Wechat to provide counseling services, with generally positive results (Chester and Glass, 2006; Lewis and Coursol, 2007; Barak et al., 2008). Specifically, for example, Nolan et al. (2011) conducted a longitudinal study where four therapists provided counseling via text messaging to 40 students over 3 years. Participants described the service as valuable to their adjustment and specifically noted the convenience and consistent availability of online support. Particularly relevant to Asian contexts, another study (Ching et al., 2009) found that Chinese immigrants in the United States reported a higher degree of comfort with text-based online counseling as compared to face-to-face.

Although online counseling appears to work for some, other studies show that it is often not the best solution. One study based in Singapore, for example, reported less effective client–counselor interactions in online settings (Kit et al., 2014): counselors reported difficulty guiding interactions, as well as a limited ability to elicit responses from clients during online sessions. Similarly, Im et al. (2007) reported that support services for cancer patients were more effective when delivered face-to-face as opposed to online. It was suggested that a lack of non-verbal cues such as tone of voice and body language limits the ability of online counselors to empathize and build rapport (Haberstroh et al., 2008). Such results are not universal, however. Wagner et al. (2014), for example, reported better outcomes 3 months after treatment for online, as compared face-to-face, clients.

Clearly, there is no argument being made here against face-to-face counseling services. It seems appropriate to consider, however, that online services may be able to fill some niche within an overall treatment space. Here, given that findings from outside Malaysia have indicated that some portion of those surveyed would be more inclined to pursue online, as opposed to face-to-face, counseling services (Borzekowski and Rickert, 2001; Bober and Livingstone, 2005; Buck et al., 2007), this study looked at relative preferences for, and the likelihood of utilizing, online as opposed to face-to-face counseling services among Malaysian university students. The research questions were:

1. Is there a specific sub-set of Malaysian university students who would prefer to utilize online as opposed to face-to-face counseling services?
2. What proportion of students report that they would only utilize online counseling?

## Method

### Participants

A paper and pencil survey was completed by 409 students from six universities across the Klang Valley region of Malaysia. The six universities included two international universities with local campuses, two private-local universities, and two local-government universities.

All participants were over 16 years, Malaysian, and current university students. Participation was voluntary, and completely anonymous. Participants did not receive any form of compensation. The participants (*n* = 409) were predominantly of Malaysian Chinese ethnicity (68.0%). The sample consisted of 41.6% (170) male participants and 58.4% (239) female participants. The age range of the participants was 16–35 years, with a median age of 20 years for both males and females; 15.2% (62) participants identified themselves as Malay; 68% (278) identified as Malaysian Chinese; 13.4% (55) identified as Malaysian Indian; and 3.4% (14) of participants identified themselves as from other Malaysian ethnic groups.

### Materials

Upon reading and signing the informed consent form, participants completed a demographic questionnaire, the Preference for Seeking Online or Face-to Face Counseling form, the Online Counseling Attitude Scale (OCAS), and the Face-to-Face Counseling Attitude Scale (FFAS). No identifying information was collected.

#### Preference for Seeking Online or Face-to-Face Counseling

Participants answered two 10-point Likert scale-type questions intended to measure the degree to which they would prefer to exclusively use either online or face-to-face counseling services. The items were: “When seeking professional help services, to what degree would you prefer to use online counseling only?” and “when seeking professional help services, to what degree would you prefer to use face-to-face counseling only?” (1 = LEAST Preferred and 10 = MOST Preferred). The variable (Pref_Only_OLC) reported in the section “Results” represents responses to the first of these questions.

#### Online Counseling Attitude Survey (OCAS)

The OCAS is a 10-item measure using a 5-point Likert-type scale (1 = Not at all and 5 = Very) (Rochlen et al., 2004). This survey measures participants’ attitudes toward online counseling. The OCAS consists of two subscales: Discomfort with Online Counseling and Value of Online Counseling. Sample items are: “I would feel uneasy discussing emotional problems with an online counselor”; “Using online counseling would help me learn about myself”. Including Asian samples, internal consistency has ranged from 0.77 to 0.90. Test–retest reliability ranged from 0.77 to 0.88 (Rochlen et al., 2004; Bathje et al., 2014).

#### Face-to-Face Attitude Survey (FFAS)

The Face-To-Face Attitude Survey (FFAS) is an adaptation of the OCAS. Consent for the adaptation was obtained and results were shared directly with Professor Rochlen, the author of OCAS. It has the same 10-items, except that the term “online counseling” was replaced with “face-to-face counseling.” It is also completed using a 5-point Likert-type scale with 1 = Not at all and 5 = Very. Sample items are – FFAS: “I would confide my personal problems in a *face-to-face* counselor”; OCAS: “I would confide my personal problems in an *online* counselor.” The coefficient alpha for the FFAS was 0.77–0.90 across several studies, which included Korean, Chinese, Indians, as well as other Asian samples (Rochlen et al., 2004).

### Procedure

In July 2015, after receiving ethical permission from the first Monash University Human Research Ethics Committee (MUHREC approval code E/2015 – CF15/1 – 2015000000), data were collected via paper and pencil questionnaires. Cluster Random Sampling technique was adopted because the population measured was too large to run a single random sampling technique. Participants completed the questionnaire in common areas on their respective campuses during lunch and snack breaks. Participants were encouraged to invite friends and other peers to complete the questionnaire in order to allow for broader sampling at minimal cost. The survey took approximately 5 min to complete.

## Results

Overall, attitudes toward both online counseling (OCAS) and face-to-face counseling (FFAS) were somewhat positive, with participants reporting a small, but statistically significant, preference for face-to-face services (OCAS Mean = 31.07, *SD* = 6.87; FFAS Mean = 33.59, *SD* = 6.64); *t*(*df*) = 7.45(408), *p* < 0.001.

In order to assess preference for online as opposed to face-to-face counseling, an additional variable “*Positive_Attitude_OLC*” was created. This variable represents OCAS minus FFAS using the average per-item score from 1 to 5. This resulted in a value ranging from -4 to +4 for each participant. A positive value for *Positive_Attitude_OLC* indicates an overall greater preference for online counseling. A negative value indicates an overall preference for face-to-face counseling. Out of 409 participants, 145, or approximately 35% of the sample, had values equal to or greater than (+)1 for this variable. In other words, on a 5-point Likert scale, 145 out of 409 participants gave preference ratings for online counseling that averaged at least 1-point higher on a scale of 1–5 than their preference ratings for face-to-face counseling.

Looking further into the strength of preference for online counseling among this subgroup, an independent-samples *t*-test was used to test whether those expressing preference for online counseling were likely to *only* use online counseling services. In other words, if online counseling services were not available, would this group be unlikely to engage in face-to-face counseling? To test this, we compared those whose *Positive_Attitude_OLC* was 1 or greater (*n* = 145) with the remainder of participants (*n* = 264) in their stated preference for using *only* online counseling (*Pref_Only_OLC*).

The results, shown in **Table 1**, indicate that those with an overall more positive attitude toward online counseling were significantly more likely to indicate that they would *only* use online counseling services. Group 1: *Positive_Attitude_OLC* ≥ 1.0 (*Pref_Only_OLC* Mean = 6.77, *SD* = 2.041). Group 2: *Positive_Attitude_OLC* < 1.0 (*Pref_Only_OLC* Mean = 5.22, *SD* = 2.383); *t*(*df*) = 6.577(407), *p* < 0.001. Thus, the group that preferred online counseling overall (145 of 409 participants, approximately 35% of the sample) had a significantly higher likelihood of reporting that they would *only* utilize online counseling services.

**Table 1:** Mean and standard deviations scores *Positive_Attitude_OLC* Group statistics.

Positive_Attitude_OLC	*N*	Mean (*SD*)	Mean difference (95% CI)	*t*-Statistic (*df*)^a^	*P*-value^a^
Pref_Only_OLC ≥1.00	145	6.77 (2.04)	1.54 (1.08, 2.00)	6.58 (407)	<0.001
<1.00	264	5.22 (2.38)			

^a^*Independent-sample t-test. Positive_Attitude_OLC; p-value < 0.001 showed statistical significance*.

## Discussion

Results indicated that, overall, Malaysian university students have a slight preference for face-to-face as compared to online counseling, although both types of counseling were viewed positively. Responses indicated that the average participant was fairly open to receiving counseling services either online or face-to-face. These results also suggest that, aside from the majority of participants who are largely open to either form of counseling, there is a sizable subset of university students (about 35% of this Malaysian sample) who would be unlikely to utilize face-to-face services but would be significantly more receptive toward the idea of online counseling. Given the stigma related to mental health issues evident in many Asian societies, as well as the inconvenience and costs involved in physically seeking help, it is not surprising that many do not seek traditional face-to-face counseling. Universities and other counseling providers, thus, may find that offering at least initial contact and basic services through digital media might encourage a significant number of clients would otherwise never engage in counseling to begin the process. Of course, the levels of service provided should be modified and possibly upgraded to face-to-face as needed. Increasing the likelihood of initial contact, and making that intial contact as painless as possible, however, may be ways in which online access can increase the reach and effectiveness of counseling services overall.

The current study has several limitations. First, the sample consisted only of university students, limiting generalizability. Second, this study focused on text-based counseling which may not be suitable for all groups. Third, this study did not directly address participants’ reasons for preferring online over face-to-face counseling or vice-versa. Future research should look at a broader sample of age groups as well as those of different ethnicities and educational levels. The practical aspects of delivering online services through various means such as, for example, the relative strengths and weaknesses of different synchronous (e.g., live chat via video, Skype, or voice) and asynchronous (e.g., text, email, messaging) methods also needs to be explored. Along with this, the reasons, reservations, and motivations underlying Malaysians’ counseling preferences and help-seeking behavior needs to be understood in more detail.

### Implications for Future Research and Counseling

Although not specifically addressed in this research, the perceived anonymity and relative convenience of online interactions likely contribute to the preference for online services expressed by many (Chang et al., 2001). Future studies need to explore the dynamics of such preferences. The more mental health providers understand the forces motivating people to seek help, or not, the better they can do in assuring that those in need of help are able to obtain it. Also, since issues related to client–counselor relationships and rapport are often cited as arguments against online counseling, future studies should focus on the processes involved in establishing rapport within mediated environments, as well as how these might be influenced by cultural contexts (Akmehmet Sekerler, 2008; Williams et al., 2009; Anthony, 2015).

In sum, about 35% of the participants in this study had a strong preference for online counseling. Considering estimates that between 9.6 and 35% of the population of Malaysia, a country of approximately 32 million people, have unmet mental health needs, innovative delivery methods such as online counseling have the potential to improve the quality of life of literally millions of people in a relatively cost-effective way (Crabtree and Chong, 2000; Chong et al., 2013). Online counseling, of course, will not ever eliminate mental health issues entirely, nor will it replace face-to-face counseling services, which continue to be the gold standard and the preferred means of treatment for most people. However, given the ongoing and apparently growing crisis related to mental well-being in Malaysia as well as other developing countries, it only makes sense that we work seriously toward delivering competent, qualified mental health services through alternative technological routes as well.

## Author Contributions

Contribution: KW, 60%; GB, 30%; CT, 5%; CW, 5%. All authors listed have made a substantial, direct and intellectual contribution to the work, and approved it for publication.

## Conflict of Interest Statement

The authors declare that the research was conducted in the absence of any commercial or financial relationships that could be construed as a potential conflict of interest.
